# A Critical Assessment of Knowledge, Attitudes, and Practices (KAP) Concerning HTLV-1 Infection Among Healthcare Professionals in Gabon

**DOI:** 10.3390/diseases14070253

**Published:** 2026-07-14

**Authors:** Hanneke Fanhole Moussonda-Mouele, Eldridge Fedricksen Oloumbou, Otniel Adjala-Ondémé, Jeordy Dimitri Engone-Ondo, Christ Eddy Ognari-Ayoumi, Moussa Yaro, Roseanne Mounanga-Mourimarodi, Krist Makosso, Ivan Sosthène Mfouo-Tynga, Augustin Mouinga-Ondémé

**Affiliations:** Unité des Infections Rétrovirales et Pathologies Associées, Centre Interdisciplinaire de Recherches Médicales de Franceville (CIRMF), Franceville BP 769, Gabon; fanholehanneke@gmail.com (H.F.M.-M.); eldridgefedricksenoloumbou@gmail.com (E.F.O.); otadjala@gmail.com (O.A.-O.); engonejordy@gmail.com (J.D.E.-O.); christayoumi@gmail.com (C.E.O.-A.); lozo.10.yaro@gmail.com (M.Y.); roseannemounanga@gmail.com (R.M.-M.); deyayemakosso@gmail.com (K.M.); tivansdavids2012@gmail.com (I.S.M.-T.)

**Keywords:** HTLV-1, knowledge, attitudes and practices, healthcare workers, Gabon

## Abstract

**Background/Objectives**: Human T-cell lymphotropic virus type 1 (HTLV-1) is an oncogenic retrovirus that infects an estimated 5–10 million people worldwide. In Gabon, despite a high seroprevalence (7.3–8.7%), documented cases of associated pathologies remain rare, suggesting significant underdiagnosis driven by a lack of clinical awareness. This study evaluated the knowledge, attitudes, and practices (KAP) concerning HTLV-1 among Gabonese healthcare professionals. **Methods**: A descriptive cross-sectional study was conducted among 392 healthcare professionals across four provinces in Gabon using a standardized questionnaire. Bivariate comparisons were performed using chi-square (*χ*^2^) and Mann–Whitney U tests, followed by multivariable binary logistic regression to identify independent predictors of HTLV-1 awareness. **Results**: Overall, 79.9% of the participants had never heard of HTLV-1. Awareness of the virus was critically low among nursing and obstetric staff (7.2%), whereas physicians demonstrated the highest level of knowledge (57.1%). In the multivariable analysis, independent predictors of higher HTLV-1 awareness included being male (aOR = 2.93; 95% CI: 1.60–5.39; *p* < 0.001), holding a university degree (aOR = 3.43; 95% CI: 1.66–6.89; *p* < 0.001), and working as a physician (aOR = 2.75; 95% CI: 1.24–6.10; *p* = 0.013). **Conclusions**: A critical HTLV-1 knowledge deficit exists among healthcare professionals in Gabon, particularly among frontline nursing staff, which likely explains the low reporting of associated diseases. Integrating HTLV-1 into medical and paramedical curricula, launching targeted educational campaigns, and implementing national screening protocols are urgently required to improve diagnosis and prevent transmission.

## 1. Introduction

Human T-cell lymphotropic virus type 1 (HTLV-1), discovered in the USA in 1980, was the first oncogenic retrovirus described in humans [1]. It is endemic in several regions globally, including southern Japan, the Caribbean, Melanesia, South America, and sub-Saharan Africa [2]. The transmission of HTLV-1 occurs primarily through three established routes: vertical transmission, particularly via prolonged breastfeeding (exceeding six months) [3,4]; sexual transmission, which occurs preferentially from males to females [4]; and parenteral transmission through contaminated blood products [5]. Additionally, zoonotic transmission via non-human primate bites has recently been reported in rural Central African populations [6].

Globally, an estimated 5 to 10 million people are infected with HTLV-1, with 5 to 10% of infected individuals developing associated pathologies [7,8]. The most prominent of these conditions are adult T-cell leukemia/lymphoma (ATL), an aggressive hematological malignancy [9], and tropical spastic paraparesis/HTLV-1-associated myelopathy (TSP/HAM), a debilitating neurodegenerative disorder affecting the lower limbs [7]. While these severe manifestations typically present in adults, other conditions, such as infective dermatitis and certain types of uveitis, are frequently associated with pediatric HTLV-1 infection [10,11].

Early HTLV-1 research in Gabon (late 1980s to mid-1990s) focused heavily on general epidemiology [12], mother-to-child transmission [3,13], and identifying associated pathologies [12,14,15,16,17]. These initial studies established an adult seroprevalence of 6.6 to 9.3% [12,14] and a pediatric seroprevalence of 2.8% [13], noting a 17.5% seroconversion risk over 4 years for children born to infected mothers [3]. Despite high seroprevalence, documented clinical cases remained rare: only five cases of TSP/HAM [12,14,15,16] and five cases of ATL [17,18] were reported nationwide between 1989 and 2005, with a likely etiologic link established between HTLV-1 and these early ATL presentations [17].

More recently, additional studies have updated the epidemiological landscape of HTLV-1 in Gabon, reporting a prevalence of 2.1% among pregnant women, 7.3 to 8.7% in the general population, 0.74% among blood donors, and 7.7% among HIV-infected individuals [5,19,20,21,22]. Furthermore, a recent clinical and epidemiological survey at the primary university hospital in Libreville, targeting patients routinely managed in the neurology, internal medicine, and dermatology departments, reported an HTLV-1 prevalence of 7.39% [23]. Among these infected individuals, only 3.7% presented with an HTLV-1-associated pathology, specifically comprising five cases of tropical spastic paraparesis/HTLV-1-Associated Myelopathy (TSP/HAM) and two cases of Adult T-cell Leukemia (ATL) [23].

To date, a total of only 17 cases of HTLV-1-associated pathologies have been officially documented in Gabon, comprising ten historical cases and seven recent ones (five TSP/HAM and two ATL) [23]. In contrast, epidemiological estimates suggest that between 16,000 and 30,000 individuals are currently living with HTLV-1 in the country [7]. Given an expected lifetime disease manifestation rate of 5% to 10%, approximately 800 to 3000 cases of HTLV-1-associated pathologies should have been identified. This profound discrepancy strongly suggests a critical lack of awareness regarding HTLV-1 among healthcare professionals, rather than the clinical rarity of these associated diseases [2,23].

This deficit positions HTLV-1 as a classically neglected infection, where gaps in clinical awareness directly translate into widespread underdiagnosis and suboptimal prevention efforts [24]. Healthcare professionals play key roles in clinical screening, patient education, and infection control, and their awareness is crucial for accurately recognizing and managing the national disease burden [25]. Consequently, this study was conducted to evaluate the knowledge, attitudes, and practices (KAP) regarding HTLV-1 among healthcare professionals in Gabon. We hypothesized that the profound underdiagnosis of HTLV-1-associated diseases is closely linked to a significant knowledge deficit among medical staff, resulting in inadequate clinical screening. Addressing this knowledge gap represents a vital step toward revealing the invisible burden of this infection and implementing effective national control measures.

## 2. Materials and Methods

### 2.1. Study Area

**Figure 1:**
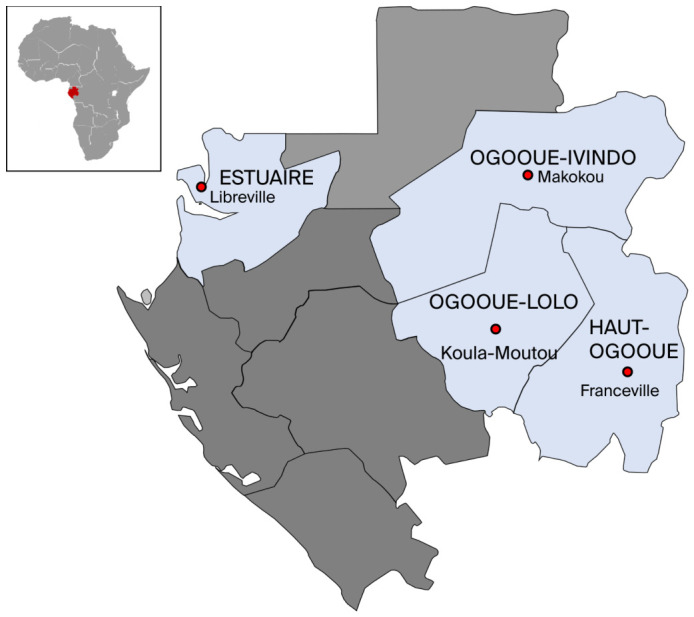
A map of Gabon highlighting the four provinces where the study was conducted: Estuaire, Ogooué-Ivindo, Ogooué-Lolo, and Haut-Ogooué.

This study was conducted across four provinces of Gabon, encompassing both urban and semi-rural areas. The selected urban sites comprised Libreville, the political capital and largest city (estimated population of 899,000 in 2025), and Franceville, the capital of the Haut-Ogooué province (population of 42,967). The semi-rural study sites included Koula-Moutou, the capital of the Ogooué-Lolo province (population of 16,222), and Makokou, the capital of the Ogooué-Ivindo province (estimated population of 13,571). Notably, Franceville, Koula-Moutou, and Makokou serve as the administrative capitals of the three provinces historically documented to have the highest HTLV-1 seroprevalence in the country (Figure 1).

### 2.2. Study Design and Population

This descriptive cross-sectional study was conducted over a two-month period, from 19 November 2024 to 18 January 2025. The investigation targeted healthcare professionals and clinical support staff working in primary care facilities or in the largest hospitals of the four selected cities.

A convenience sampling approach was used to enrol available personnel during the study period. To ensure statistical power, the minimum required sample size was calculated using Cochran’s formula. Assuming an expected HTLV-1 knowledge level (*p*) of 50% (to yield the maximum required sample size), a 5% margin of error (d), and a 95% confidence level (Z = 1.96), a minimum of 384 participants was required. Our final sample of 392 participants exceeded this threshold and was deemed statistically adequate [26]. The participating hospitals across the study sites are detailed in Table 1.

The study population comprised healthcare professionals and clinical support staff, including medical, nursing, technical, and auxiliary personnel, actively working across the various departments of the participating hospitals. Conversely, personnel from administrative and accounting departments, as well as temporary trainees, were systematically excluded from the study.

### 2.3. Data Collection and Questionnaire

A structured questionnaire was developed based on a comprehensive review of the HTLV-1 literature and existing KAP studies [27,28,29]. The tool was divided into three primary sections: (1) sociodemographic characteristics, including age, gender, place of residence, occupation, years of service, level of education, marital status, and hospital of employment; (2) HTLV-1 knowledge, focusing on modes of transmission, prevention strategies, clinical manifestations, diagnosis, and treatment options; and (3) attitudes and practices, assessing participants’ professional attitudes and current clinical practices regarding HTLV-1.

To ensure face validity and contextual clarity within the Gabonese healthcare system, the preliminary questionnaire was rigorously reviewed by the senior co-authors, leading to minor adjustments in the phrasing of clinical items. Additionally, the instrument was pre-tested on a small pilot sample of healthcare workers (*n* = 20), who were excluded from the final analysis, to confirm readability and comprehensibility. Reliability analysis demonstrated acceptable internal consistency of the questionnaire, yielding a Cronbach’s alpha coefficient of 0.72.

Strict criteria were established to evaluate response accuracy. Specifically, participants were required to correctly identify the virus as transmissible and acknowledge the current absence of specific curative treatments or vaccines. Regarding clinical manifestations, a correct response necessitated the identification of two hallmark pathologies: adult T-cell leukemia/lymphoma (ATL) and HTLV-1-associated myelopathy/tropical spastic paraparesis (TSP/HAM). Prevention knowledge was validated if at least two preventive methods were stated. For diagnostic proficiency, responses were deemed correct if at least one recognized biological technique was identified, such as serological screening (enzyme-linked immunosorbent assay, ELISA) confirmed via Western blot/INNO-LIA or molecular biology (PCR). Finally, a transmission score ranging from 0 to 4 was calculated. Knowledge levels were categorized as “good” (four modes identified), “fairly good” (three modes), “passable” (two modes), and “poor” (one or zero modes).

The purpose and methodology of the study were initially presented during meetings with department heads and hospital personnel across all participating facilities. Following these sessions, each department head was provided with a set of questionnaires to distribute to their respective staff for self-administration of the survey.

### 2.4. Statistical Analysis

The collected data were consolidated into a central database and pre-processed using R software (version 4.4.2). This data-cleaning and curation phase involved formatting standardization, management of missing entries, and the generation of composite analytical variables. Once data preparation was complete, all subsequent statistical analyses were performed using JAMOVI software (version 2.3.28).

Descriptive statistics were initially computed to characterize the study variables using frequencies and percentages for qualitative variables. For quantitative variables, distributions were summarized using measures of central tendency and dispersion, including the mean, standard deviation (SD), median, and range.

In the inferential analysis, categorical variables were compared using the chi-square (*χ^2^*) test. Continuous variables were analyzed using the Mann–Whitney U test after confirmation of non-normal distribution via the Shapiro–Wilk test. A *p*-value less than 0.05 (<0.05) was considered statistically significant.

Finally, multivariable analysis using binary logistic regression was performed to identify sociodemographic factors independently associated with HTLV-1 knowledge. The initial model included variables demonstrating statistical significance in the univariate analysis alongside those deemed clinically relevant. Model fit and explanatory performance were assessed using pseudo-R^2^ statistics (McFadden, Cox and Snell, and Nagelkerke). Multicollinearity among independent variables was evaluated using variance inflation factors (VIFs) and tolerance values, with a VIF less than 5 (<5) and a tolerance greater than 0.5 considered acceptable (tolerance > 0.5). The results were expressed as adjusted odds ratios (aORs) accompanied by their corresponding 95% confidence intervals (95% CIs). All analyses were two-tailed, with statistical significance defined as a *p*-value less than 0.05 (*p* < 0.05).

### 2.5. Ethical Considerations

This study was approved by the Direction of the International Centre for Medical Research (CIRMF). Prior to enrollment, the objectives and methodology of the investigation were thoroughly explained to all potential participants. Written informed consent was subsequently obtained from each participant involved in the study.

## 3. Results

### 3.1. Demographic Characteristics

A total of 392 healthcare workers participated in the study. The population was predominantly female (73.7%, 289/392), while 26.3% (103/392) were male. Most participants (77.2%) were from urban areas, primarily Libreville (39.7%, 156/392) and Franceville (37.5%, 147/392). Regarding institutional distribution, the largest shares of respondents were employed at the teaching hospitals: CHUAB (29.8%, 117/392) and CHUL (27.3%, 107/392). The mean age of the participants was 39.7 years (SD ± 9.7), ranging from 20 to 64 years. The median age was 38.5 years, with an interquartile range (IQR) of 32–48 years. The most represented age group was 30–39 years at 33.9% (133/392). Educational attainment was high, with nearly two-thirds of the cohort (62.5%, 245/392) holding a university degree. In terms of professional roles, nursing and obstetrical staff were the most common (63.5%, 249/392), and a plurality of participants reported a length of service between 0 and 5 years (38.5%, 151/392).

### 3.2. Knowledge, Attitudes, and Practices (KAP) Regarding HTLV-1

Overall awareness of the virus was low, with nearly 80% (313/392) of participants reporting they had never heard of HTLV-1. Analysis of the subgroup of 79 participants who reported prior awareness of the virus revealed significant associations with specific demographic and institutional factors.

Regarding the sources of information, university training was the primary means of knowledge acquisition, cited by 14% (55/392) of all participants. Online resources were utilized by 3.8% (15/392) of participants. Specific knowledge regarding transmission was poor: only 16.6% (65/392) of the surveyed personnel correctly identified that the virus is transmissible (Table 2). When asked about the specific routes of infection, only 1.5% (6/392) of participants successfully identified all four modes of HTLV-1 transmission (Table 3).

With regard to the clinical manifestations, more than 90% (90.3%, 354/392) of healthcare workers were unaware of how this infection presents, whereas fewer than 5% of participants (4.6%, 18/392) provided correct answers by mentioning one or more associated pathologies. Regarding diagnosis, only 4.1% (16/392) knew how it is established, 6.1% (24/392) gave incorrect answers, and nearly 90% (89.8%, 352/392) admitted they did not know. When questioned about the availability of therapeutic options, only 4.6% (18/392) answered correctly, 4.8% (19/392) gave an inaccurate response, and 90.6% (355/392) were completely unaware of whether a treatment exists. This knowledge gap extended to vaccination; only 5.9% (23/392) correctly stated that no vaccine is currently available. Furthermore, only 7.9% (31/392) of participants were aware of prevention methods. Despite these gaps, the vast majority of the participants (94.1%, 369/392) expressed a strong desire for further education regarding the virus, citing the media (69.4%, 272/392) as the most preferred communication channel.

Bivariate analysis revealed that prior knowledge of HTLV-1 was significantly associated with gender, level of education, and profession (all *p* < 0.001). Specifically, awareness was notably higher among males (37.9%, 39/103) than females (13.8%, 40/289). Educational attainment also played a critical role; 26.9% (66/245) of university-educated professionals reported prior knowledge of the virus, in contrast to only 9.4% (12/127) of those with a high school education. Regarding occupational categories, physicians demonstrated the highest level of awareness (57.1%, 24/42), followed by other healthcare workers (41.4%, 12/29) and medical laboratory staff (36.8%, 25/68). Conversely, nursing and midwifery staff exhibited the lowest awareness level at just 7.2% (18/249). Other sociodemographic factors, including age group, years of service, and hospital affiliation, did not show any statistically significant association with HTLV-1 knowledge (*p* > 0.05) (Table 4).

Multicollinearity diagnostics revealed no significant collinearity among the independent variables, with all variance inflation factor (VIF) values approaching 1.0 (VIF < 5) and tolerance values exceeding 0.90. The multivariable logistic regression model demonstrated acceptable explanatory performance, with a McFadden’s R^2^ of 0.08, a Cox and Snell R^2^ of 0.08, and a Nagelkerke R^2^ of 0.13.

In the multivariable logistic regression model, gender, level of education, and profession were confirmed as strong independent predictors of HTLV-1 awareness (Table 5). Males had nearly three times higher odds of being aware of the virus than females (aOR = 2.93; 95% CI: 1.60–5.39; *p* < 0.001). Similarly, healthcare professionals with a university degree were over three times more likely to possess prior knowledge of HTLV-1 than those with a high school education or less (aOR = 3.43; 95% CI: 1.66–6.89; *p* < 0.001). Furthermore, profession emerged as a major determinant: when compared to medical laboratory staff (reference group), physicians were significantly more likely to be aware of the infection (aOR = 2.75; 95% CI: 1.24–6.10; *p* = 0.013). In contrast, nursing and midwifery staff exhibited significantly lower odds of HTLV-1 awareness (aOR = 0.13; 95% CI: 0.06–0.27; *p* < 0.001). Years of service remained non-significant in the adjusted model.

## 4. Discussion

In the medical world, HTLV-1 infection remains a largely invisible condition, as the vast majority of carriers are asymptomatic. Clinical manifestations are relatively rare, with only 2 to 5% of infected individuals developing associated pathologies over their lifetime [4,7,8]. Although the WHO has recognized this infection as a public health concern, a comprehensive global program dedicated to combating HTLV-1 remains absent [8]. Nonetheless, the virus is highly endemic in Gabon, with prevalence rates ranging from 7.3 to 8.7% in the general population. This high endemicity, coupled with its association with various locally documented diseases and the lack of systematic screening protocols for high-risk groups, such as blood donors and pregnant women, underscores the critical need for heightened awareness among healthcare workers [2,30].

This study is the first to formally assess the knowledge, attitudes, and practices (KAP) of healthcare workers regarding HTLV-1 within an endemic area in Gabon. The finding that nearly 80% of healthcare workers had never heard of HTLV-1 is alarming, given that these professionals represent the frontline defence for the prevention and management of this infection. This deficit raises a fundamental question: why is baseline knowledge so critically low in a highly endemic country? This paradox may be largely attributed to the “institutional invisibility” of the virus. Unlike HIV or malaria, which benefit from robust national control programs and systematic screening guidelines, HTLV-1 lacks dedicated public health policies in Gabon. This structural deficiency results in an absence of a clinical culture surrounding the virus, rendering it invisible to both policymakers and frontline practitioners. Consequently, this severe lack of awareness directly contributes to why HTLV-1 remains underestimated as a public health threat in the country [31].

This observation aligns with broader research highlighting the pivotal role of healthcare workers in public health outcomes. Indeed, two studies conducted in Italy corroborate the idea that the level of knowledge among healthcare professionals is a critical determinant of the general population’s acceptance of and adherence to preventive measures. Research on vaccine hesitancy, particularly regarding influenza and COVID-19, demonstrates that explicit recommendations from healthcare professionals are key factors in encouraging the adoption of preventive measures [24,25]. In the context of HTLV-1, where prevention relies on targeted screening (particularly among blood donors and pregnant women) and specific advice on transmission routes, a knowledge deficit among healthcare personnel results in a lack of systematic and appropriate advice. Without a thorough understanding of the infection, healthcare workers cannot fulfil their role as primary vectors of awareness, thereby perpetuating the cycle of transmission and underdiagnosis in the population.

This systemic neglect is particularly evident when awareness is analyzed across occupational categories. Our results reveal a striking professional disparity: while 57.1% of physicians were aware of the virus, knowledge plummeted to just 7.2% among nursing and obstetric staff. This gap is directly tied to institutional training; although HTLV-1 epidemiology is covered in university-level medical programs, it is systematically omitted from the curricula of nursing and paramedical schools in Gabon. This result is consistent with the limited data available from other endemic regions, such as a study in Brazil, where 70% of nursing students reported being unaware of the infection [32]. This occupational disparity also aligns with recent international findings; a 2025 cross-sectional study by Salimi et al. exploring HTLV-1 insights among healthcare providers similarly demonstrated that while physicians achieved the highest scores, nurses and laboratory technicians exhibited critical knowledge and practice gaps [33].

The scarcity of similar KAP studies on HTLV-1, even within other endemic regions, is itself a testament to the global neglect of this virus. This neglect is largely tied to the virus’s prolonged incubation period, as most carriers remain asymptomatic throughout their lives, rendering early detection difficult. Consequently, institutional training programs prioritize pathogens with high immediate morbidity, such as malaria and HIV. Furthermore, when an HTLV-1-related disease occurs (such as ATL or HAM/TSP), it is frequently misdiagnosed or “masked” by broader clinical labels, such as lymphomas or myelopathies of unknown origin. This diagnostic challenge, as underscored by the vast disparity between estimated prevalence and the number of documented clinical cases reported in Gabon, directly contributes to severe underreporting of the true disease burden [23].

Analogous research in other clinical domains highlights similar gaps in the surveillance and clinical recognition of certain diseases when relying solely on population-based measures. For instance, studies on vaccination coverage among splenectomized patients [34,35] and immunity gaps against poliovirus [36] indicate that vulnerable cohorts remain inadequately protected and monitored in the absence of targeted awareness campaigns and structured protocols. This parallel strongly supports the notion that the discrepancy between the high prevalence of HTLV-1 and the low number of clinically reported cases in Gabon is driven by structural, rather than biological, factors. Ultimately, this gap reflects a systemic failure of clinical recognition, rooted in a deficit of knowledge, sub-optimal attitudes, and inadequate practices among healthcare professionals.

The finding that only 1.5% of participants possessed a complete understanding of all modes of transmission is a critical concern. Given that mother-to-child transmission via prolonged breastfeeding is one of the primary routes of viral propagation, this knowledge deficit poses a substantial public health risk. A lack of clinical awareness on this specific point directly compounds the risk of vertical transmission, a risk that has been successfully mitigated in other endemic nations. For instance, the implementation of nationwide prenatal screening and rigorous breastfeeding counselling has been well-documented to significantly reduce the incidence of this infection in Japan [37,38].

The profound lack of knowledge regarding clinical manifestations (fewer than 5% of correct answers) and diagnosis (only 4.1% proficiency) represents a major barrier to effective disease management. Although clinical cases of HTLV-1-associated malignancies, such as adult T-cell leukemia/lymphoma (ATLL), have been documented in Gabon, these conditions are highly susceptible to being misdiagnosed or confounded with other pathologies in the absence of provider awareness. These conditions are highly susceptible to being misdiagnosed or confounded with other pathologies in the absence of provider awareness. This diagnostic gap inevitably leads to delayed or inappropriate therapeutic interventions [23].

This study identified a highly significant association between gender and baseline knowledge of the virus. Multivariable analysis confirmed that this link remains independent of confounding variables such as age or seniority, with men being nearly three times more likely than women to be aware of HTLV-1 (aOR 2.93). This asymmetry does not reflect discrepancies in individual cognitive competence, but rather the sociological stratification of healthcare professions in Gabon. The nursing and midwifery sectors, which displayed the lowest awareness rates, are predominantly staffed by women. Conversely, medical and laboratory professions, which have greater exposure to scientific literature, have historically comprised a higher proportion of men [39]. Notably, this gender discrepancy appears to be highly context-dependent. A 2026 study by Letafati et al. assessing HTLV-1 awareness among healthcare workers in Tehran found no statistically significant gender-based differences in knowledge, confirming that the gap observed in our cohort reflects specific regional professional dynamics rather than a universal trend [40].

Furthermore, the significant association between a higher education level and knowledge of the virus aligns with academic expectations. Healthcare professionals holding a university degree (aOR 3.43) are better equipped to independently access international scientific literature and utilize research databases. Consequently, these highly educated Gabonese health professionals are far more likely to have been exposed to specialized courses on tropical infectious diseases during their university curricula.

A powerful lever for future intervention strategies is the participants’ overwhelmingly positive attitude, with more than 90% of healthcare workers expressing a strong desire for further education regarding HTLV-1 infection. Furthermore, the declared preference for mass media and digital channels as the primary means of information sources provides strategic guidance for upcoming public health policies. Consequently, it is crucial that future awareness campaigns intentionally leverage these modalities, including television, radio, and online platforms, to maximize reach and impact among both healthcare workers and the general public.

The limitations of this study are primarily methodological, pertaining to sample representativeness and the inherent constraints of self-reported data. First, self-report bias may have influenced the findings; despite the anonymous design, participants might have consulted external resources, such as the internet, while self-administering the questionnaire. This potential social desirability bias may have led to an overestimation of baseline knowledge levels. Second, a well-documented gap exists between self-reported knowledge and actual clinical practice. Third, because our sample was drawn exclusively from major healthcare facilities in urban and semi-rural centres, the findings may not be fully generalizable to professionals operating in remote rural areas or smaller, peripheral clinics. Finally, because systematic HTLV-1 screening has not yet been implemented in Gabon, we could not correlate provider knowledge with routine testing rates or biological surveillance data. Consequently, the logical next step is not merely to repeat KAP surveys, but to integrate knowledge assessments into the rollout of pilot screening protocols, particularly for pregnant women and blood donors. This integration would provide an objective framework to evaluate how targeted training interventions directly translate into increased case detection and epidemiological reporting.

## 5. Conclusions

This study is the first to formally assess the knowledge, attitudes, and practices (KAP) regarding HTLV-1 among healthcare professionals in Gabon. Our findings demonstrate that, despite the high endemic prevalence of HTLV-1 and documented clinical diagnoses of associated pathologies such as TSP/HAM and ATL, the vast majority of healthcare workers remain unaware of this infection. This profound knowledge deficit likely drives the chronic underdiagnosis and underreporting of HTLV-1 cases nationwide, highlighting an urgent need for targeted public health policies and institutional training.

To effectively bridge these gaps and improve national health outcomes, we propose a multi-faceted public health framework. First, foundational medical and paramedical curricula must be systematically updated to incorporate mandatory modules on HTLV-1 epidemiology, clinical diagnostics, and preventive strategies. Second, healthcare institutions should implement mandatory continuing education programs to upskill current practicing staff. Third, targeted awareness campaigns utilizing mass media and digital channels should be deployed to educate both healthcare workers and the general population. Crucially, public health authorities must establish systematic, routine screening protocols for high-risk groups, specifically pregnant women and blood donors, integrated with specialized provider training. Finally, Gabon should officially observe International HTLV-1 Day every November 10th, aligning it with established national campaigns for malaria and HIV. This institutional advocacy should be coordinated in close collaboration with national professional bodies and associations, including the physician, midwife, and nursing boards, alongside international development partners.

Finally, future research should expand into a comprehensive, nationwide, multi-center KAP study to validate these findings across all nine provinces of Gabon. Additionally, longitudinal intervention studies are warranted to evaluate the real-world effectiveness of targeted educational programs in improving clinician diagnostic accuracy and patient outcomes. Implementing these multi-level strategies is essential to optimizing the diagnosis, clinical management, and prevention of HTLV-1, ultimately mitigating the overall burden of this neglected retroviral infection across the country.

## Figures and Tables

**Table 1:** Hospitals selected in various cities.

Province/City	Hospital Name(s)
Haut-Ogooué/Franceville	Centre Hospitalier Universitaire Amissa BONGO (CHUAB) and Hôpital de l’Amitié Sino-Gabonaise (HASG)
Estuaire/Libreville	Centre National d’Hémodialyse (CNH), Centre Hospitalier Universitaire de Libreville (CHUL) and Hôpital d’Instructions des Armées d’Akanda (HIAA)
Ogooué-Lolo/Koula-Moutou	Centre Hospitalier Régional Paul MOUKAMBI (CHRPM)
Ogooué-Ivindo/Makokou	Centre Hospitalier Régional Omar BONGO ONDIMBA (CHROBO) and Centre de Traitement Ambulatoire (CTA)

**Table 2:** Knowledge about HTLV-1 among participants (N = 392).

Aspects of Knowledge	Correct		Incorrect		Don’t Know	
	*n*/N (%)					
Is it transmissible?	65/392	16.6	01/392	0.3	326/392	83.2
What are the clinical manifestations?	18/392	4.6	20/392	5.1	354/392	90.3
Is there a treatment for this infection?	18/392	4.6	19/392	4.8	355/392	90.6
Is there a vaccine for this infection?	23/392	5.9	06/392	1.5	363/392	92.3
How to prevent this infection?	31/392	7.9	02/392	0.5	359/392	91.6
How is HTLV-1 diagnosed?	16/392	4.1	24/392	6.1	352/392	89.8

**Table 3:** Knowledge about transmission modes among participants (N = 392).

Level of Knowledge	*n*/N	(%)
Well (4/4 modes)	6/392	1.5
Fairly (3/4 modes)	31/392	7.9
Passable (2/4 modes)	16/392	4.1
Weak (1/4 modes)	10/392	2.6
Don’t know	329/392	83.9

**Table 4:** Sociodemographic characteristics and their association with prior knowledge of HTLV-1 (N = 392).

Sociodemographic Characteristics	*n*/N	(%)	*p*-Value
Gender			
Male	39/103	37.9	
Female	40/289	13.8	
Age Groups (Years)			0.979
20–29	10/63	15.9	
30–39	30/133	22.6	
40–49	24/107	22.4	
≥50	12/71	16.9	
NA	3/18	16.7	
Level of education			<0.001
Primary school	0/1	0.0	
High school	12/127	9.4	
University	66/245	26.9	
NA	1/19	5.3	
Profession			<0.001
Medical laboratory staff	25/68	36.8	
Nursing and obstetric staff	18/249	7.2	
Physicians	24/42	57.1	
Other healthcare workers	12/29	41.4	
NA	0/4	0.0	
Years of Service			0.504
0–5	32/151	21.2	
6–10	10/41	24.4	
11–15	9/35	25.7	
16–20	11/55	20	
>20	12/69	17.4	
NA	5/41	12.2	
Hospitals			0.844
CHUAB	23/117	19.7	
CHUL	19/107	17.8	
HIAA	11/37	29.7	
HASG	9/32	28.1	
CHRPM	8/41	19.5	
CHROBOM	7/45	15.6	
CTAM	1/3	33.3	
CNH	1/10	10	

CHUAB: Centre Hospitalier Universitaire Amissa BONGO; CHROBOM: Centre Hospitalier Régional Omar BONGO ONDIMBA; HASG: Hôpital de l’Amitié Sino-Gabonaise; CHUL: Centre Hospitalier Universitaire de Libreville; HIAA: Hôpital d’Instruction des Armées d’Akanda; CHRPM: Centre Hospitalier Régional Paul MOUKAMBI; CNH: Centre National d’Hémodialyse; CTAM: Centre de Traitement Ambulatoire de Makokou; NA: Not Available.

**Table 5:** Multivariable analysis of knowledge about HTLV-1.

Variables	*n*/N	aOR	95% CI	*p*-Value
Gender				
Female	40/289	Ref.	–	–
Male	39/103	2.93	1.60–5.39	<0.001
Level of Education				
High school	12/127	Ref.	–	–
University	66/245	3.43	1.66–6.89	<0.001
Profession				
Medical laboratory staff	25/68	Ref.	–	–
Nursing and obstetric staff	18/249	0.13	0.06–0.27	<0.001
Physicians	24/42	2.75	1.24–6.10	0.013
Other healthcare workers	12/29	0.79	0.31–2.00	0.62
Years of Service				
0–5	32/151	Ref.	–	–
6–10	10/41	1.12	0.48–2.61	0.79
11–15	9/35	1.34	0.57–3.16	0.51
16–20	11/55	0.97	0.45–2.09	0.93
>20	12/69	1.12	0.39–1.79	0.64

## Data Availability

All data supporting the reported results of this study are included in the manuscript. Additional information may be available upon request from the corresponding author.
